# Intake of silica nanoparticles by giant lipid vesicles: influence of particle size and thermodynamic membrane state

**DOI:** 10.3762/bjnano.5.256

**Published:** 2014-12-23

**Authors:** Florian G Strobl, Florian Seitz, Christoph Westerhausen, Armin Reller, Adriano A Torrano, Christoph Bräuchle, Achim Wixforth, Matthias F Schneider

**Affiliations:** 1 Lehrstuhl für Experimentalphysik I, Universität Augsburg, 86159 Augsburg, Germany; 2 Nanosystems Initiative Munich NIM, Schellingstr. 4, 80799 München, Germany; 3 Institut für Physik, Universität Augsburg, 86159 Augsburg, Germany; 4 Department of Chemistry and Center for NanoScience (CeNS), University of Munich (LMU), 81377 Munich, Germany; 5 Department for Mechanical Engineering, Boston University, Boston, MA 02215, USA

**Keywords:** cells, endocytosis, engulfment, fission, gel phase, giant unilamellar lipid vesicles (GUV), lipid membranes, liquid phase, nanoparticle, phosphocholines, uptake, vesicles, wrapping

## Abstract

The uptake of nanoparticles into cells often involves their engulfment by the plasma membrane and a fission of the latter. Understanding the physical mechanisms underlying these uptake processes may be achieved by the investigation of simple model systems that can be compared to theoretical models. Here, we present experiments on a massive uptake of silica nanoparticles by giant unilamellar lipid vesicles (GUVs). We find that this uptake process depends on the size of the particles as well as on the thermodynamic state of the lipid membrane. Our findings are discussed in the light of several theoretical models and indicate that these models have to be extended in order to capture the interaction between nanomaterials and biological membranes correctly.

## Introduction

**Figure 1:**
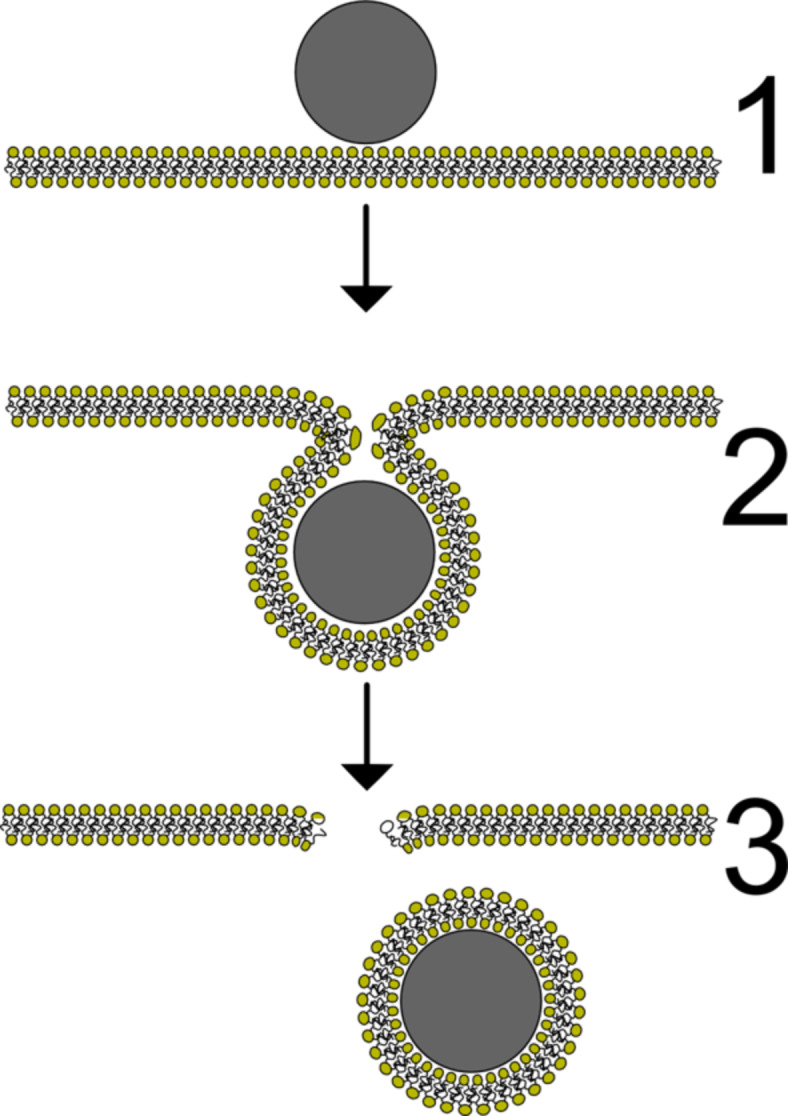
An endocytosis-like uptake of particles involves three major steps: adhesion (1), engulfment (2), and fission (3). During the last process a membrane defect is induced, which will heal over time.

Nanomaterials gain more and more importance in different industrial and scientific branches and the rising probability of accidental exposure of humans and their environment to nanoparticles gave rise to the development of the relatively new research field of nanotoxicity [1–4]. The uptake of nanoparticles by living cells and the related risks play a crucial role in these areas. The high efficiency of this uptake in many cases recommends the application of nanoparticles as drug carriers or contrast agents [5–6]. While it was shown that very small nanoparticles can directly penetrate cell and model membranes [7–8], particles significantly larger than the membrane thickness (3–4 nm) are usually taken up by endocytosis [9–10]. The physical aspects of an interaction between cell membranes and nanoparticles are still not well understood. Especially the dependence of the uptake efficiency on the particle size is still an important topic [11–12]. Usually, a complex cell machinery is involved in endocytosis. However, particle uptake including the engulfment by the membrane has also been reported for red blood cells, which are known for not possessing such a machinery [13]. This indicates that endocytosis-like particle uptake can be driven by physical interactions between cargo and cell membrane. The investigation of simplified model systems thus offers a possibility for an understanding of these processes on a theoretical physical base. In this context, the terminology “endocytosis-like” is not supposed to imply the involvement of external energy supplies. It rather refers to the vast amount of (theoretical) models describing the process of endocytosis by changings, for instance, in curvature, bending, stress, due to the interaction with proteins (e.g., clathrin coats) or the induction of membrane asymmetry [14–15]. The three main steps of such an uptake are depicted in Figure 1: adhesion to the membrane, bending of the membrane until the full encapsulation of the cargo and detachment of a vesicle from the membrane by a fission process. Mechanical aspects of such a colloid–membrane interaction are treated by several theoretical models.

A simple, purely mechanical picture of such an interaction involves at least three mechanical parameters: the adhesion energy per unit area *g*_ad_, the bending stiffness of the membrane *κ* and its surface tension σ. In the limit of large particles the bending energy can be neglected, because the membrane curvature necessary for an envelopment is small. Helfrich described the bending energy per unit area of a fluid membrane by means of its principal curvatures 1/*R*_1_ and 1/*R*_2_ [16]:

[1]
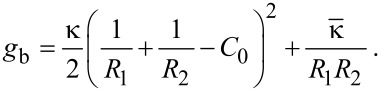


The last term, containing the saddle splay modulus 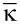 is often neglected, as it can be shown that during morphological transitions of a membrane without a change in topology this term is constant [17]. However, for the case of a complete uptake involving fission, this contribution has to be considered. Starting from Equation 1, one can analyze the competition between membrane bending stiffness and particle–membrane adhesion and deduce a critical radius *r*_crit_ [18]. A spherical adhering particle will only be engulfed by the membrane if its radius *r* fulfills the condition

[2]
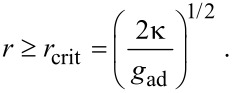


Typical values are *κ* = 10^−19^ J for fluid membranes [19] and *g*_ad_ = 1 mJ/m^2^ (see below). This results in *r*_crit_ = 14 nm. Hence, the bending stiffness of the membrane should be considered for particles in the nano-regime.

As soon as the membrane under observation exhibits a finite surface tension, its area compressibility modulus *g*_ten_ has to be considered as well, since membrane area is consumed during the wrapping process. Dietrich et al. introduced a model for vesicle–particle interaction in the large particle limit in which the wrapping process is mainly limited by the membrane tension [20]. This model is confirmed by experiments with latex beads in the micrometer-range. However, as mentioned before, the influence of the bending energy cannot be neglected for particles in the nano-regime.

**Figure 2:**
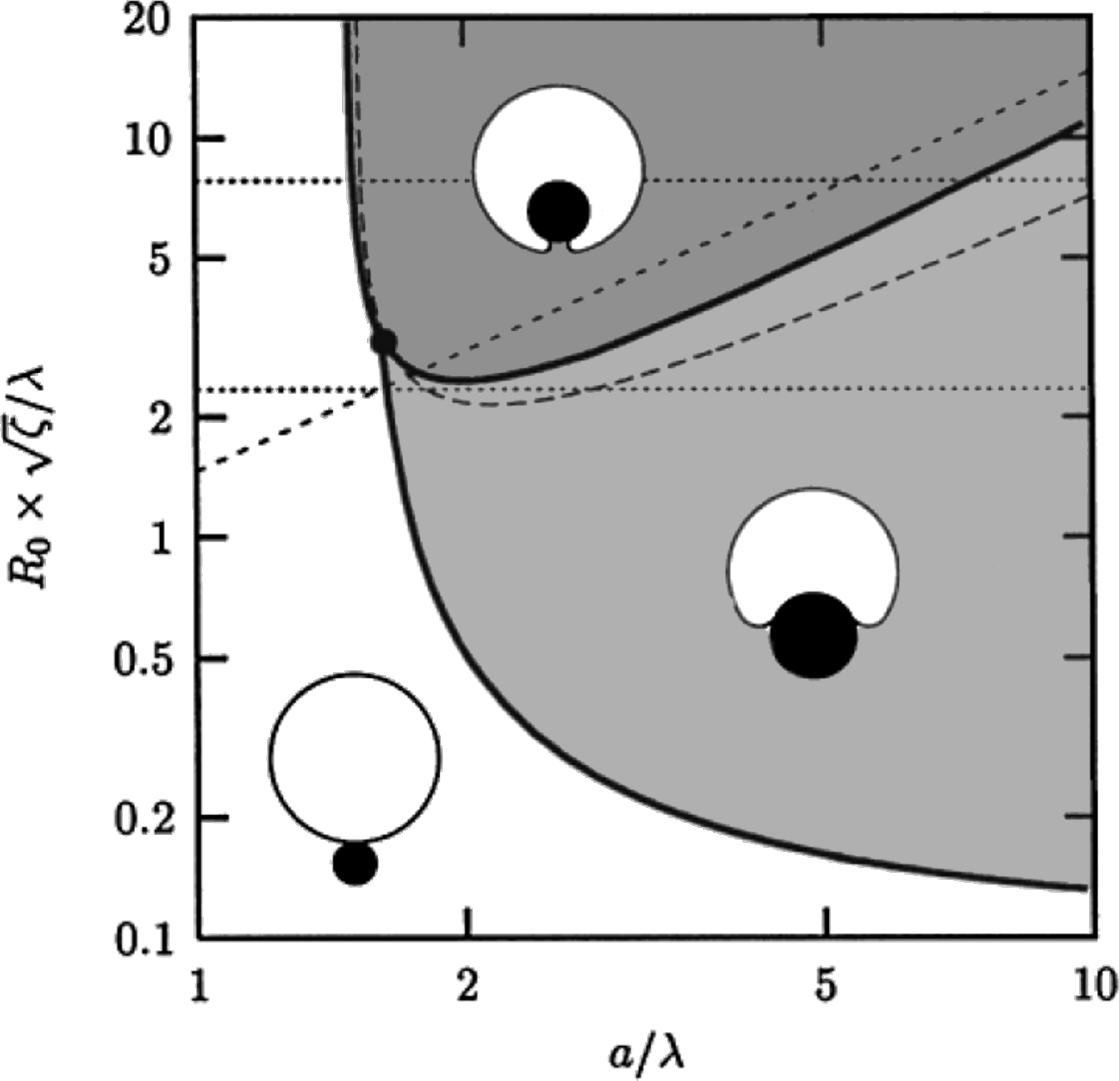
Phase diagram describing the interaction of particles with a spherical vesicle with initially zero tension. The parameters are here defined as follows: *R*_0_ denotes the initial vesicle radius; *a* corresponds to the particle radius *r*; 
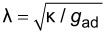
; *ζ* = *g*_ad_/*g*_ten_. In short, very small particles will not be wrapped at all due to the bending resistance (white). The wrapping of big particles is rather limited by membrane tension (light grey). Only for low vesicle tension and sufficiently big particles full wrapping is possible (dark grey). Reprinted with permission from [21]. Copyright 2002 American Chemical Society.

Deserno and Gelbart finally published a model considering both, tension and bending and, additionally, the line tension arising from the bending energy stored in the neck region of a membrane bud [21]. The results of this model are nicely described by the phase diagram depicted in Figure 2. It involves three different phases: no interaction, partial wrapping, full ingestion.

Moreover, in some studies the interaction of membranes and colloids and cooperative phenomena due to membrane mediated interactions of membrane bound particles is investigated [22–24]. Although there are some experimental reports on the interaction of nanoparticles with lipid bilayers [20,25–26], there are only few studies systematically testing the above mentioned theoretical models. To our knowledge, there are only very few publications showing an effective endocytosis-like particle accumulation by lipid vesicles [27–28]. The vesicles used are usually of a size significantly below that of a cell and/or exhibit mechanical properties different from typical cell-sized giant vesicles. As a consequence, the interaction of single or a few particles with a vesicle of comparable size is observed. However, a living cell is typically some orders of magnitudes larger than nanoparticles and, thus, can easily interact with many particles simultaneously. Another aspect that did not gain a lot of attention so far is the mutual interplay of the adsorption behavior of nanoparticles and the phase state of membranes. In [29], for example, it was shown that the phase transition temperature of lipid membranes changes upon the interaction with silica nanoparticles. Our own research group recently found a significant and systematic dependence of this phase transition shift on the particle size, e.g., the bending radius of the membrane [30], which we explained by employing a simple model based on a combination of Landau’s theory of phase transitions and Helfrich’s bending energy approach. It appears obvious that phase separation and phase transitions play an important role in the function of cell membranes in general [31] and for membrane traffic in particular [32]. These effects are usually discussed in terms of the role of phase separations for cell signaling and protein recruiting. However, lipid membranes also change their mechanical and morphological properties during thermodynamic phase transitions, rendering *κ* and 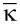 thermodynamic quantities, which depend on the membrane state. Therefore, the investigation of nanoparticle–membrane interactions in the light of the thermodynamic state of the membrane can yield important insights into the processes involved in cellular particle uptake. To our knowledge, experiments in this direction are very rare, to date. Some examples of previous work, however, will be addressed in the discussion part of this manuscript.

In the following, we present results about the interaction of silica nanoparticles in contact with giant unilamellar phospholipid vesicles (GUVs). In this simple system, all major steps of a particle uptake, as depicted in Figure 1 are found. It will be shown that this behavior is clearly dependent on the particle size and the phase state of the membrane. Our experimental findings will be discussed in the light of the theoretical models mentioned above. We conclude that the existing theories are not sufficient to describe the observed phenomena and we will present a simple approach for the description of a membrane interacting with more than one particle.

## Results

The simple experimental procedure is described below in the corresponding section and Figure 9. Basically, silica particles are brought into contact with GUVs residing at the bottom of a temperature controlled chamber. The concentration of particle surface area (see Experimental section for definition) is *C*_s_ ≈ 10 m^2^/L in all experiments. The interaction of particles and vesicles is then observed by fluorescence microscopy.

### Uptake of particles induced by double layer force

**Figure 3:**
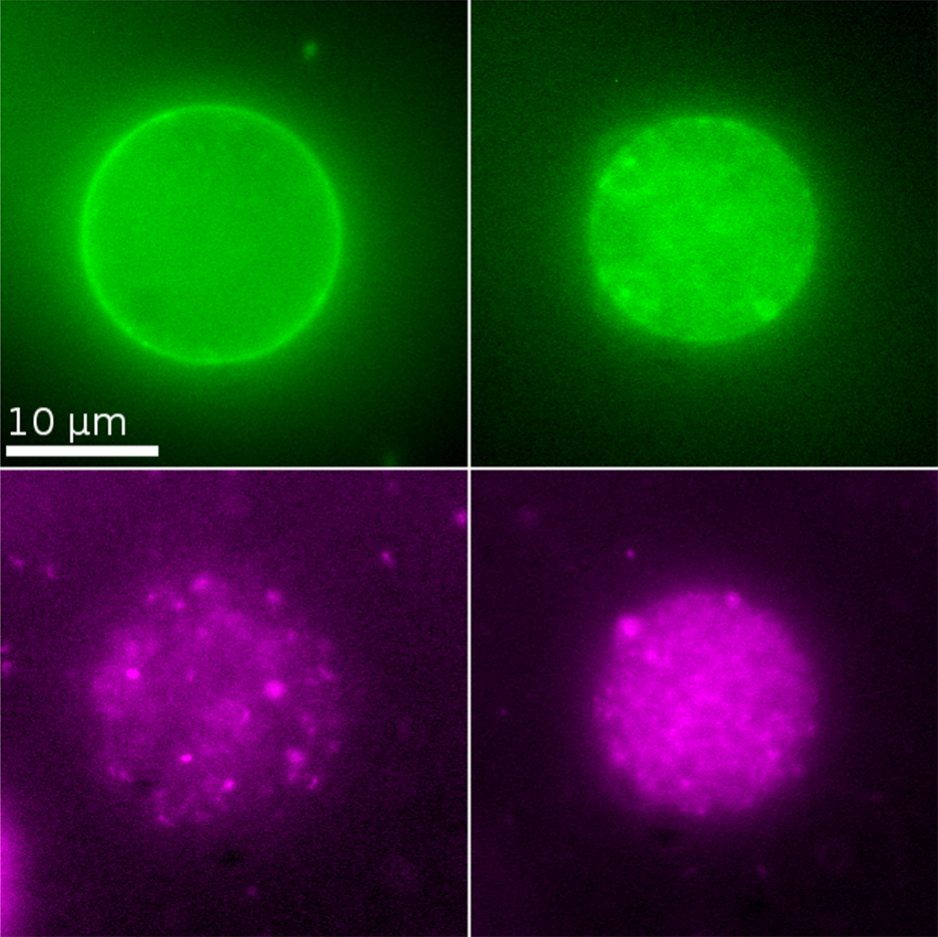
1,2-dioleoyl-*sn*-glycero-3-phosphocholine (DOPC) vesicle (green), 1 min (left) and 10 min (right) after the incubation with nanoparticles (*r* = 42 nm, magenta). Obviously, vesicle membrane is consumed while particles are internalized.

To enable an uptake as described before, there must be a sufficient attractive force between the particle and the membrane. As shown in [33], the interaction between a neutral (i.e., zwitterionic) lipid bilayer and negatively charged silica surface is repulsive in pure water but attractive in phosphate buffered saline. The authors also give a plausible theoretical explanation for this finding by taking into account the double layer interaction in this asymmetrical system. This force can be repulsive in dilute salt solutions (regime of constant charge of the silica) or attractive at high salt concentration (regime of constant potential). The threshold salt concentration for strong attraction is found to be of the order of 10 mM. Indeed, in our experiments no significant adhesion of nanoparticles at the vesicle surface could be observed up to a critical ionic strength of the medium 10 mM ≤ *I*_crit_ ≤ 20 mM. Above this critical value, particles with sufficient small radii adhere to the membrane and massive particle uptake takes place. Figure 3 illustrates this situation.

During the uptake process, the vesicle radius, i.e., the membrane area shrinks continuously, whereas the intravesicular particle concentration increases. Furthermore, the particles become visible after internalization in the optical channel of the membrane label. They diffuse freely inside the vesicle membrane. This indicates that the particles are engulfed by the membrane and that the uptake process involves fission of the membrane sheath from the vesicle membrane like in Figure 1. This conclusion is in agreement with other studies [27–28].

To guarantee attractive interaction, all further experiments were carried out at an ionic strength of *I* = 32 mM. Unfortunately, it is not easy to give a good estimate for the adhesion energy per unit area *g*_ad_ under these conditions, since none of the two above limits apply. Nevertheless, following [33] and assuming interaction at constant potential (high salt limit), an upper limit for the double layer interaction *g*_dl_ can be approximated by the Hogg–Healy–Fuerstenau equation:

[3]
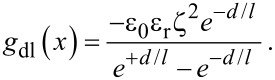


**Figure 4:**
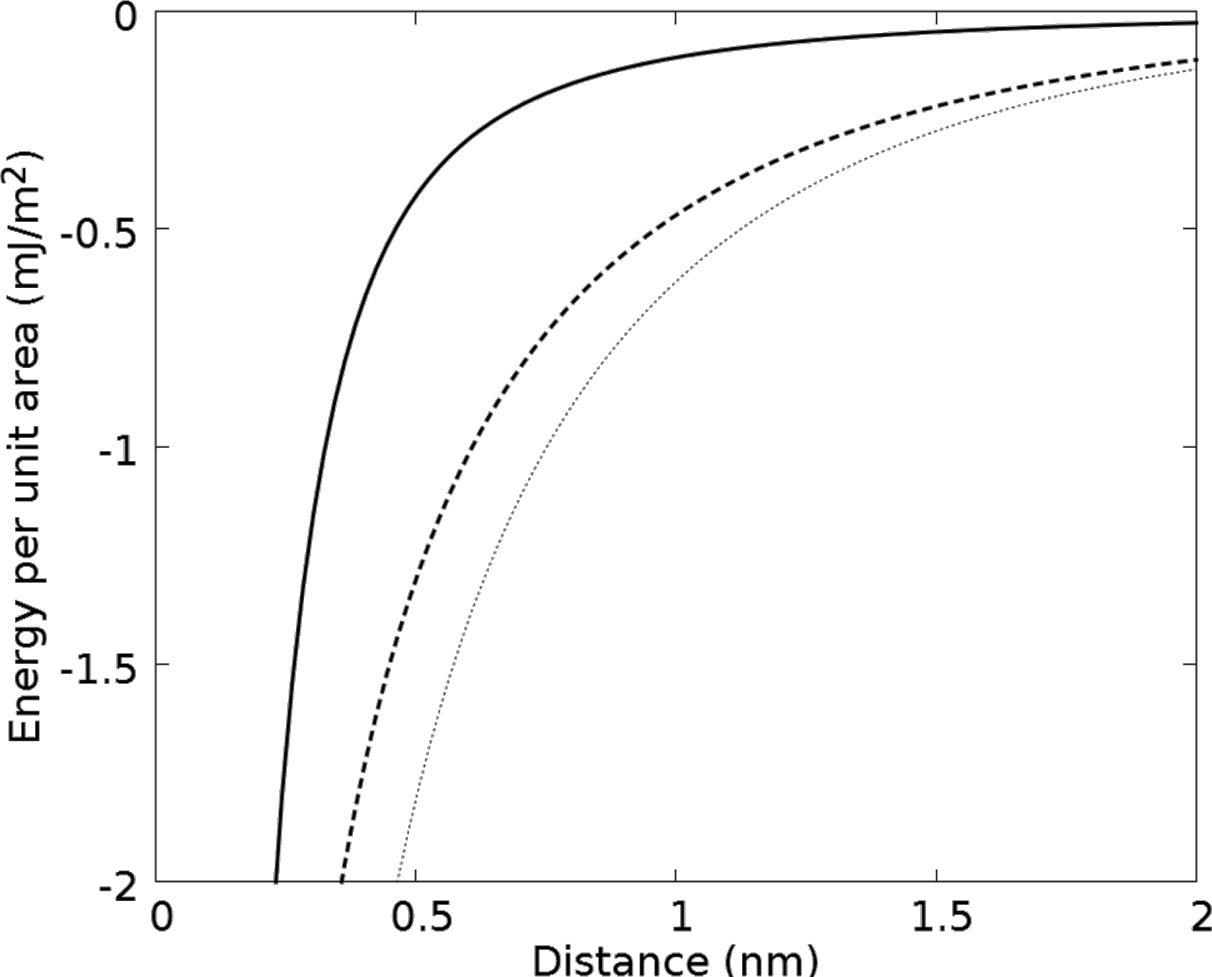
Expected van der Waals (solid line) and double layer (dashed line) binding energies as a function of the particle–membrane distance. The double layer interaction dominates the system for the relevant separation distances. Additionally the solution of the Hogg–Healy–Fuerstenau-equation for a cationic particle with *ζ* = +30 mV and a membrane with *ζ* = −30 mV at *l* = 0.77 nm (dotted line) is given.

Here, *d* is the distance from the silica surface, ε_0_ε_r_ the dielectric constant of the medium and *l* the Debye length. The *ζ*-potential is inserted for the relevant surface potential, as also proposed in [33]. In Figure 4, *g*_dl_ is plotted for *ζ* = −50 mV and *l* = 1.72 nm and ε_r_ = 79, corresponding to our experimental conditions. Additionally, an approximation for the non-retarded van der Waals interaction is given (see Equation 2 in [33]). Assuming a surface to surface distance of 0.5 nm ≤ *d* ≤ 1 nm, *g*_a_ ≈ *g*_dl_ ≈ −1 mJ/m^2^ can serve as an upper limit for the attractive adhesion energy.

### Rising membrane tension upon particle uptake

Assuming a process as described in Figure 1, the uptake of one particle with radius *r* will consume a membrane patch with a surface area of *A*_p_ = 4*πr*^2^. If the vesicle volume would stay constant, the uptake of particles would stop at latest as soon as the surface tension of the vesicle σ exceeds the adhesion energy per unit area:

[4]
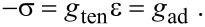


Here, *g*_ten_ denotes the area compressibility modulus and ε the relative area excess:

[5]
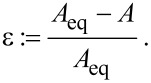


**Figure 5:**
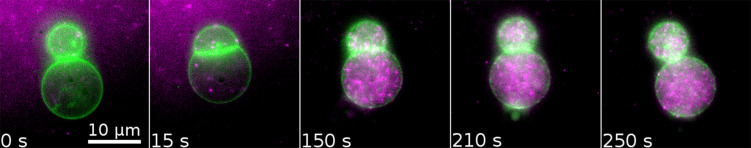
Time series of two DOPC vesicles in close contact. Upon the uptake of particles, initially a sudden, strong adhesion is induced, followed by a successive return to spherical shape. At 15 s the maximum adhesion area is achieved.

*A* is the actual surface area and *A*_eq_ the area the lipids would cover at equilibrium with zero surface tension. Estimating this limit for our system with *g*_ad_ = −0.5 mJ/m^2^ and *g*_ten_ = 200 mN/m leads to a value ε = 0.25%. However, the vesicles typically shrink substantially with a loss of surface area over 50%. Thus, some of the inner medium has to escape from the vesicle during particle uptake. One hypothesis that will be discussed later is that the opening of pores is promoted by the fission process (see Figure 1). These pores are thought to be stable enough to maintain a finite surface tension during the uptake process by the release of inner medium. The rising tension of the vesicle membrane can be followed nicely in some experiments (Figure 5) as follows: If two vesicles touch each other, these vesicles initially show a sudden adhesion, followed by a slower separation, once a significant number of nanoparticles has entered.

This behavior can be explained by tension-induced adhesion of vesicles as predicted in [34]. According to this model, vesicles with negligible surface tension exhibit thermal undulations. These undulations induce a mutual repulsive force and prevent adhesion. However, once the vesicle tension rises, these undulations are suppressed and adhesion can take place (step 1–2 in Figure 5). Upon ongoing particle uptake, the surface tension then rises further and finally forces the vesicles back into spherical morphology. This leads to two statements:

- The vesicles exhibit significant thermal undulations before particle uptake.
- The “equilibrium” surface tension during particle uptake is high enough to exceed the mutual adhesion energy between vesicles. That is, the equilibrium excess surface area is significantly negative: ε < 0.

### Influence of particle size and membrane state

**Figure 6:**
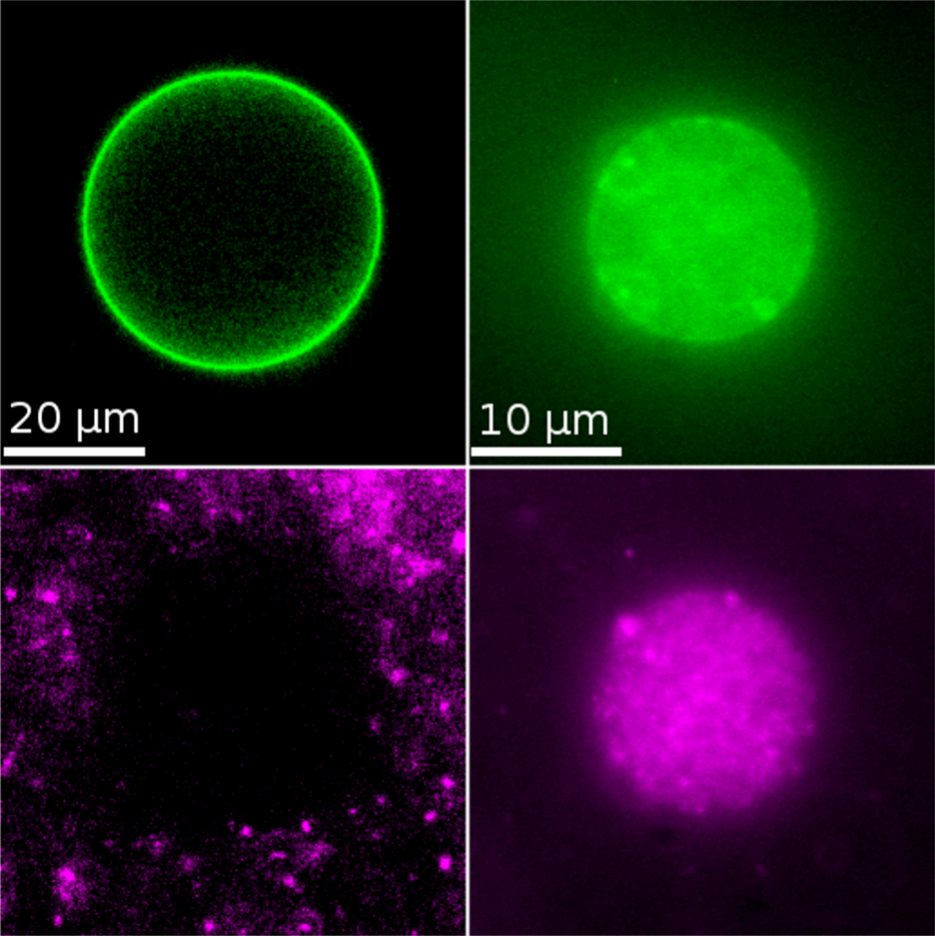
Size dependence for fluid phase vesicles. Left**:** A DOPC vesicle (green) after incubation with 123 nm particles (magenta) for 15 min. There are no signs of particle uptake. Right**:** The vesicle from Figure 3 after 10 min incubation with 42 nm particles. The contrast between these two systems is obvious.

The described behavior was observed for silica particles with radii of *r* = 42 nm as well as *r* = 11 nm in several independent experiments. In contrast, particles with *r* = 123 nm do not show any distinct interaction with vesicles in the fluid phase. Figure 6 shows a DOPC vesicle after incubation with such large particles for 30 min. Neither permanent adhesion, nor uptake of particles are observed for this system at the chosen experimental conditions. The same behavior is observed for 1,2-dimyristoyl-*sn*-glycero-3-phosphocholine (DMPC) vesicles at temperatures above the phase transition temperature (data not shown).

Very small particles with *r* = 11 nm are taken up with approximately the same efficiency as the particles with *r* = 42 nm, even though the significantly weaker *ζ*-potential of these particles *ζ* = −27 mV suggests a smaller adhesion strength *g*_ad_ compared to the two larger particle species with *ζ* = −50 mV.

**Figure 7:**
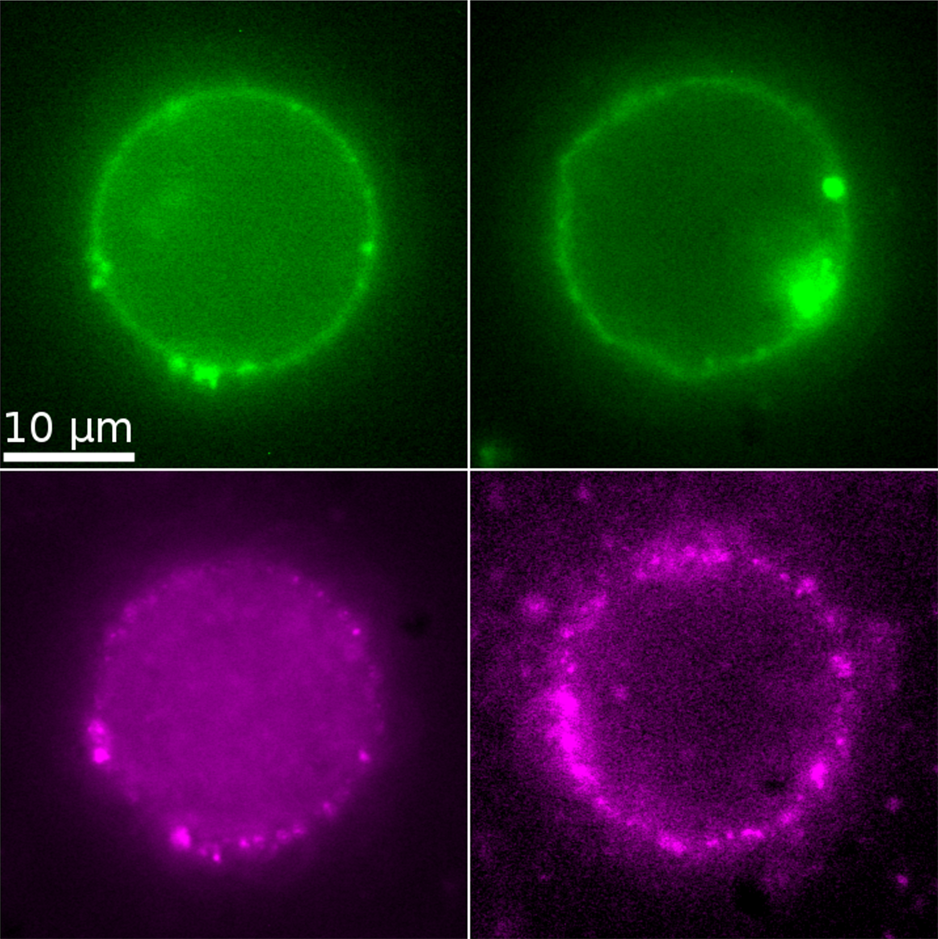
Gel phase vesicles (green) after incubation with particles (magenta). Left: 42 nm particles. The uptake behavior here is comparable to the fluid phase situation. Right: 123 nm particles. In contrast to fluid phase vesicles (see Figure 6), the particles adhere strongly to the membrane and some are internalized. However most particles remain bound to the membrane.

The uptake of particles was also tested for GUVs in their gel phase state. The investigated lipid compositions were DMPC at a temperature of 15 °C and an equimolar mixture of DMPC and 1,2-dipalmitoyl-*sn*-glycero-3-phosphocholine (DPPC) at room temperature (phase transition regime 30–35 °C as confirmed by DSC measurements). Here, all particles, including those with *r* = 123 nm are taken up as described above. However, more particles stay attached to the membrane after adhesion. This effect is particularly pronounced for the large 123 nm particles (Figure 7). In this case, only few particles diffuse within the membrane, but a very high particle load of the membrane can be observed.

Assuming a quasi equilibrium with σ = const during particle uptake, the rate of particle uptake can be deduced from the reduction of the surface area of the vesicle *A*. For this purpose, the surface area of the vesicle was monitored over time as described in the Experimental section. In Table 1 we compare the derived rates of relative area loss 1/*τ* for gel and fluid phase for the different particle species. Except for the particles with *r* = 123 nm, all uptake rates are in the same order of magnitude. In most cases, this should represent a good measure of the particle uptake rate. However, for the case of the 123 nm particles and gel phase vesicles this has to be questioned, since an adhesion with only partial wrapping will also consume membrane area. Thus the actual uptake rate will be lower than the simple area-loss argument suggests.

**Table 1:** Rates of relative area loss 1/τ for different membrane-particle systems.

	gel phase (10^−3^ s^−1^)	fluid phase (10^−3^ s^−1^)

*r* = 11 nm	−3 ± 1	−3 ± 1
*r* = 42 nm	−5 ± 3	−5 ± 4
*r* = 123 nm	approx. −2^a^	no uptake

^a^Most particles are bound to the membrane.

## Discussion

Why are small particles preferred for an uptake over large ones and why is an uptake of large particles only possible for gel phase vesicles? This question will be discussed here seperately with regard to membrane tension and to bending stiffness. Later on we comment on the biological relevance of our findings.

### Surface tension during an uptake of many particles

As described in the previous section, there is a finite surface tension counteracting particle uptake. This leads to the phase boundary between full wrapping and partial wrapping in Figure 2. Ignoring the influence of the bending energy, this effect was investigated in [20] and Deserno and Gelbart give an approximation for the threshold ratio between particle radius *r* and vesicle radius *R*, depending on the relative area excess ε [21]:

[6]
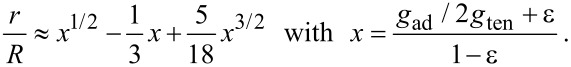


In Figure 2, the short dashed line indicates this threshold, assuming only one single particle in contact with a vesicle with excess area ε = 0.

Here we want to give a tentative analysis for an interaction of many particles with such a vesicle. If we assume a vesicle with constant area, each particle will contribute to a decrease in ε(*N*). In that case, at some number of internalized particles *N*_thr_, the surface tension will reach a threshold where no further particle uptake is possible. Recalling Equation 5, ε(*N*) can be calculated as

[7]
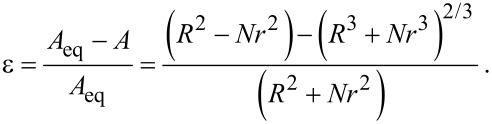


**Figure 8:**
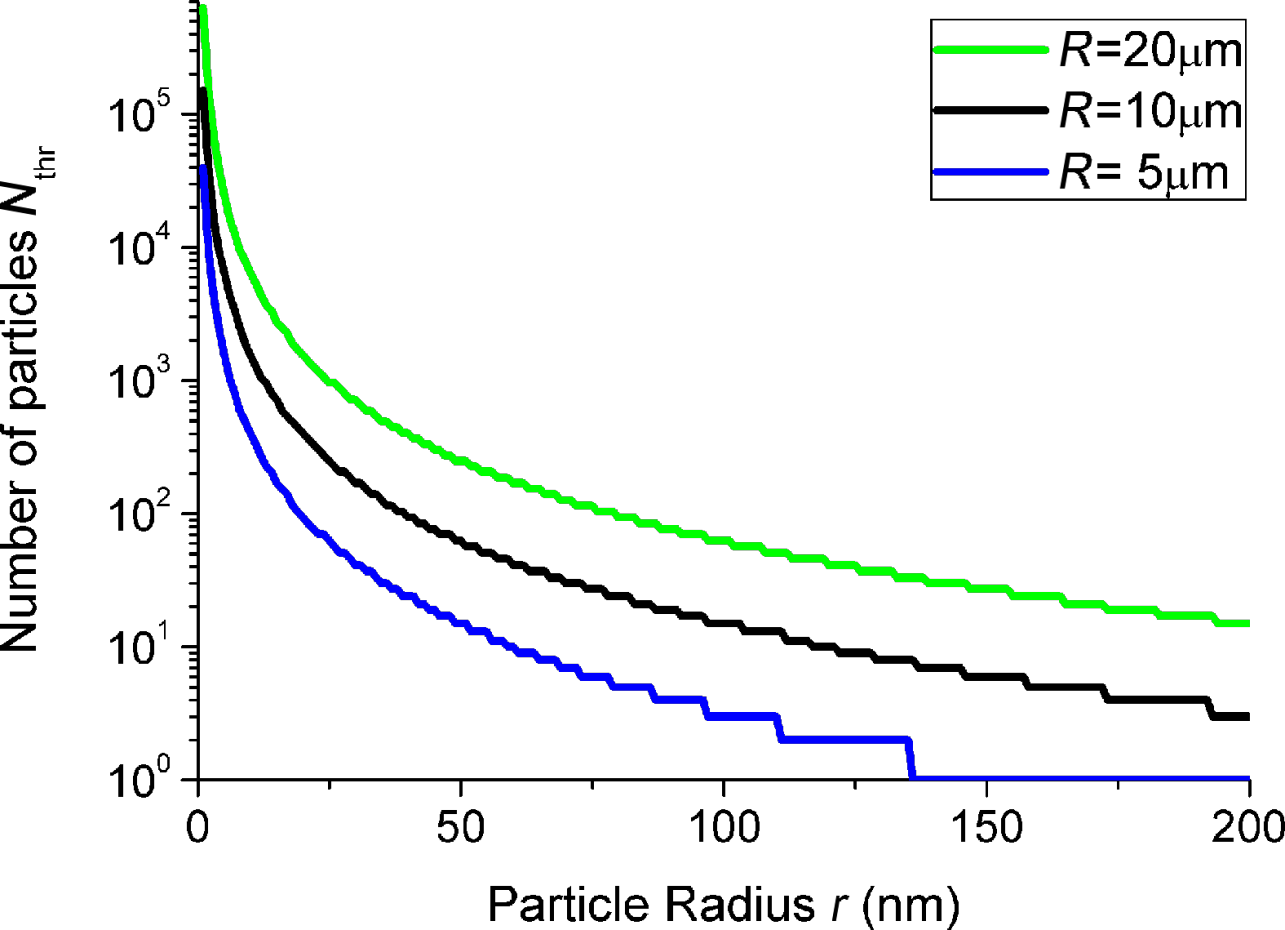
Determination of the threshold number of particles *N*_thr_ for an uptake without volume loss. The different curves represent different initial vesicle radii *R*. *g*_ad_ = −0.5 mJ/m^2^ and *g*_ten_ = 200 mN/m were chosen as before.

*R* denotes the vesicle radius before uptake and r the particle radius. Inserting this into Equation 6 delivers a relation between the ratio *r*/*R* and the maximum number of particles that can be internalized *N*_thr_, assuming a non-leaking vesicle. *N*_thr_(*r*) was found numerically for different vesicle radii *R* and plotted in Figure 8. *N*_thr_(*r*) of course depends on the ratio *g*_ad_/*g*_ten_ and on the initial vesicle radius *R*.

One can deduce following information from this simple approach: In the case of 123 nm particles, the uptake of only a few particles already induces a surface tension that is sufficient for suppressing a further uptake. For the other particle species, this value is orders of magnitude higher. However, a model assuming a constant surface area can certainly not explain the observed massive particle uptake for all sizes.

If we now assume the simple case that each fission event induces a pore with characteristic size and opening time, the permeability of the vesicle will scale linearly with *N*. Hence, it seems very plausible that during an uptake of small particles a vesicle is able to release enough volume to hold the surface tension at a sub-threshold equilibrium value, whereas this is not possible during the uptake of large particles, during which only very few pores are produced. The role of pores in fission processes and the release of cargo from giant vesicles was also the subject of earlier works in our group [35–36].

Due to the non-zero lateral shear resistance of gel-phase membranes, the relaxation time for the healing of defects will be much longer as compared to liquid membranes. Hence, induced pores remain stable over a longer time and thus lead to a reduced membrane tension. Along that line, membrane tension could serve as one explanation for the observed trends. However, this model is of hypothetical nature so far and has to be tested further. Experiments monitoring the development of surface tension during the uptake process are subject to current experiments and will make such a test possible. Also, this model neglects many important additional aspects. A deeper analysis of the observed effects and the interaction of particles with membranes in general would demand theoretical models including bending stiffness, surface tension and thermal undulations. One should also be aware that at the observed uptake rates of up to 3000 particles/s, it is likely that several particles are taken up simultaneously and cooperative effects can occur. One question along that line is the energy balance and mutual influence between different pores or fission sites in the membrane. Another possibility to keep in mind is the uptake of particle clusters instead of single particles. We will discuss this in the next subsection. A thorough treatment of these aspects would go beyond the scope of this present work and will be discussed elsewhere.

### Bending energy

According to those models including bending energy, there exists a lower threshold value for the particle radius, below which a wrapping of particles should be hindered by the bending rigidity (see Figure 2). Inserting typical values (*κ* = 1.5·10^−20^ J [19], *g*_ad_ = −0.5 mJ/m^2^) into Equation 2 leads to 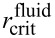 ≈ 8 nm for DOPC membranes. For gel-phase membranes, *κ* is typically one order of magnitude higher than for fluid membranes. For DMPC and DPPC *κ* ≈ 10^−18^ J has been determined experimentally [37–38]. The corresponding critical radius is 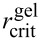 ≈ 45 nm. Hence, the bending energy that has to be overcome would prevent the uptake of particles with *r* = 42 nm and *r* = 11 nm. This prediction is obviously disproved by our results. One explanation for that could be that Helfrich’s theory for the bending energy (Equation 1) and, as a consequence, also Equation 2 are, strictly speaking, only correct for fluid membranes and might not be applicable for the gel-phase case. Another important aspect could be that the higher bending rigidity of gel-phase membranes will effectively prevent thermal fluctuations of the membrane. Hence, the repelling undulation forces will vanish in this case, which leads to stronger effective adhesion as compared to fluid membranes. In that way, high bending stiffness can contribute indirectly to stronger adhesion between membrane and particle. Undulation forces can be expected to influence large particles stronger than smaller ones, since a flat surface in contact with a flat membrane will “feel” all undulation modes, whereas very small particles will not be influenced by fluctuations with long wavelengths. Anderson et al. measured much stronger adhesion forces for gel-phase than for fluid membranes [33]. In [39] it was shown that nanoparticles accumulate preferably into the gel phase of phase-separated GUVs. Both authors mention undulation forces as one possible reason for the observed effects. However, the typical strength of undulation forces is similar to that of the van der Waals interaction [40] and hence rather too weak to explain the observed uptake for gel-phase vesicles.

So far, the influence of the bending energy rather opposes the observed trends regarding particle size and membrane phase state. However, for a process including fission, the Gaussian bending energy has also to be taken into account. There are not many measurements for the saddle splay modulus 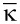 available, but the occurrence of a fission processes and other indications suggest negative values especially for ordered phase lipids [28,30,41–42]. This in turn, can significantly facilitate fission processes. A possibility that should not be overseen is a particle-induced phase separation in the mixed lipid membranes used. As shown earlier, phase separation can trigger budding and fission processes [43–44].

Finally, one should be aware of the fact that very small particles tend to cluster before an uptake into vesicles [27–28]. Thus, the effective radius being relevant for the uptake process might be larger than the single particle radius in the case of the 11 nm particles. Such clustering processes can occur due to membrane-mediated interactions, provided the bending imprint of the particles is strong enough [22,25,45]. Our data, however, do not indicate an internalization of particle agglomerates.

Taken together, further experiments will be necessary to exclude these mentioned effects. The relevance of the membrane bending stiffness for the observed phenomena still remains questionable. Especially for the large 123 nm particles, it will probably play a minor role and membrane tension will be the limiting factor for particle uptake.

### Biological relevance

Along with a long list of publications [35,43,46] the model presented here demonstrates the wealth of phenomena present already in systems of fairly “simple” composition. This wealth arises from physical interactions and possible thermodynamic changes of the lipid membrane, which can result in drastic alterations of its physical properties (e.g., bending stiffness, permeability and spontaneous curvature). Even though the model system that was investigated here is quite distinct from a real cell membrane we want to point out that it provides strong evidence that such aspects are highly important for cellular particle uptake and must not be ignored in biology. To elaborate, we would like to comment shortly on the energetic circumstances in biologically relevant cell–particle interactions compared to those in the model.

As a first example we want to highlight the uptake of cationic particles. The highly efficient uptake of those particles compared to their anionic counterparts [47–48] is not surprising if one takes into account the typically negative net charge of cell membranes. *ζ*-potential values of −20 mV for HeLa cells and −30 mV for red blood cells were measured [49]. The dotted curve in Figure 4 shows the expected electrostatic force between a cationic particle with *ζ* = +30 mV and a cell membrane with *ζ* = −30 mV in a medium with an ionic strength of *I* = 160 mM. The physical forces in this realistic scenario are significantly stronger than those that are necessary for an uptake in our model system (see Figure 4). In the case of anionic or neutral particles, electrostatic forces will, of course, not be sufficient for an uptake. However, binding of proteins can provide strong adhesion. Scavenger receptors are known to mediate the uptake of a big diversity of negatively charged cargo. For instance, Lunov et al. [50] show that scavenger receptor A plays a crucial role in the uptake of 20 nm iron oxide particles by macrophages. They derive from their data, that up to 20 receptors are involved in the uptake of one particle. If one assumes a binding energy of 15·*k*_B_*T* for each receptor, which is a reasonable strength for specific binding [51], the density of adhesion energy provided by the receptors is approximately 1 mJ/m^2^. This is in good agreement with the forces in our model system.

Of course, in a biological system, active mechanisms play a key role in cellular uptake. But these examples show that the significance of physical interactions might often be underestimated. This is also indicated in several studies, showing striking similarities between the uptake of membrane-active macromolecules by cells on the one hand and passive model systems on the other hand [52–53].

One very important implication of our data is that even the fission of loaded vesicles can be achieved without the help of active proteins such as dynamin. This aspect is usually not regarded in the discussion about particle uptake. We assume the distinct mechanical and thermodynamic properties of lipid bilayers to play a major role here. Further studies should investigate the influence of local particle-induced phase separation on this process, which earlier findings indicate [30,35,44]. This is particulary important in light of the fact that biological membranes reside near phase transitions [54], a phenomenon so far unexplained but continuously and increasingly observed. In particular, near phase transitions external changes (e.g., the adsorption of molecules or particles) can trigger enormous changes.

## Conclusion

In summary, by employing a simple model system consisting of GUVs and silica nanoparticles, we have shown that unspecific adhesion can lead to a massive uptake of particles. The uptake process exhibits the major steps of an endocytosis, including fission, i.e., the separation of engulfed particles from the membrane. The particle uptake induces substantial membrane tension but is not limited by the associated negative area excess of the GUVs. The process occurs for liquid-phase as well as for gel-phase vesicles and small particles are internalized more effectively than large particles. The latter are only internalized into gel-phase vesicles under the chosen experimental conditions. These findings are somewhat surprising from the point of view of mechanical models for membrane–particle interaction. However, these models describe the interaction of single particles with a membrane and neglect important thermodynamic aspects. We discussed that the existence of fission-induced pores could explain the successive relief of membrane tension that is necessary for a continuous particle uptake. The thermodynamic state of the membrane can influence the adhesion strength between particle and membrane and the typical relaxation time of membrane defects. Both parameters are very important for uptake processes. The facts, that massive internalization of particles can be driven by unspecific interaction of lipid membranes and that this is dependent on the phase state of the membrane are highly relevant for biological systems. We would like to point out that the presented process shows intriguing similarities with the process of endocytosis and is described by similar theoretical concepts even though it does not involve active mechanisms or energy consumption. It has been shown that nanoscale objects can be internalized independent from complex cell machineries and that lipid domains (rafts) play a crucial role in cellular uptake mechanisms [13,32,53]. An understanding of the unspecific physical aspects of membrane–particle interactions is of vital importance for a discussion of these findings.

## Experimental

1,2-dioleoyl-*sn*-glycero-3-phosphocholine (DOPC), 1,2-dimyristoyl-*sn*-glycero-3-phosphocholine (DMPC) and 1,2-dipalmitoyl-*sn*-glycero-3-phosphocholine (DPPC) dissolved in chloroform were purchased from Avanti Polar Lipids (Alabaster, Alabama, USA), 3,3′-ditetradecyloxacarbocyanine (DiOC_14_) from Biotium Inc. (Hayward, CA, USA), Na_2_HPO_4_, NaH_2_PO_4_, sucrose and D-(+)-glucose monohydrate from Merck (Darmstadt, Germany). For aqueous solutions ultrapure water (pure Aqua, Germany) with a specific resistance ≥18 MΩ was used.

GUVs were prepared by electroformation as described for the first time by Angelova et al. [55]. In short, lipids in the desired ratio and 0.05 mol % of the fluorescent marker DiOC_14_ were mixed in chloroform and spread onto indium tin oxide (ITO)-coated glass slides. The solvent is thoroughly removed through vacuum evaporation. For the swelling procedure, a chamber was assembled from two of the slides and filled with 150 mM sucrose solution. An AC-voltage (*f* = 10 Hz, *E*_eff_ = 0.6 V/mm) was applied for several hours. The temperature was hold well above the highest phase-transition temperature of the used lipids. The osmolarity of the used solutions was measured with an Osmomat 030 (Gonotec, Germany). Silica nanoparticles were synthesized as described elsewhere [56]. In the case of the particles with *r* = 42 nm Cy5 was used as label instead of perylene. Particle size distributions were characterized by SEM and the *ζ*-potentials measured with a Zetasizer (Malvern, USA). These data can be found in Table 2.

**Table 2:** Physical parameters for the used silica nanoparticles.

	size (nm)	*ζ*-potential (mV)

11 nm particles	11 ± 3	−27 ± 10
42 nm particles	42 ± 10	−52 ± 18
123 nm particles	123 ± 13	−50 ± 8

The medium used for the experiments was phosphate buffered glucose solution. The buffer was prepared from Na_2_HPO_4_ and NaH_2_PO_4_ and adjusted to pH 7. The osmolarity was adjusted by addition of glucose to be equal to the osmolarity of the sucrose solution inside the GUVs. This is to prevent osmotic tension of the vesicle membrane at the beginning of an experiment. The colloidal stability of the particles under these conditions was confirmed by dynamic light scattering (DLS).

**Figure 9:**
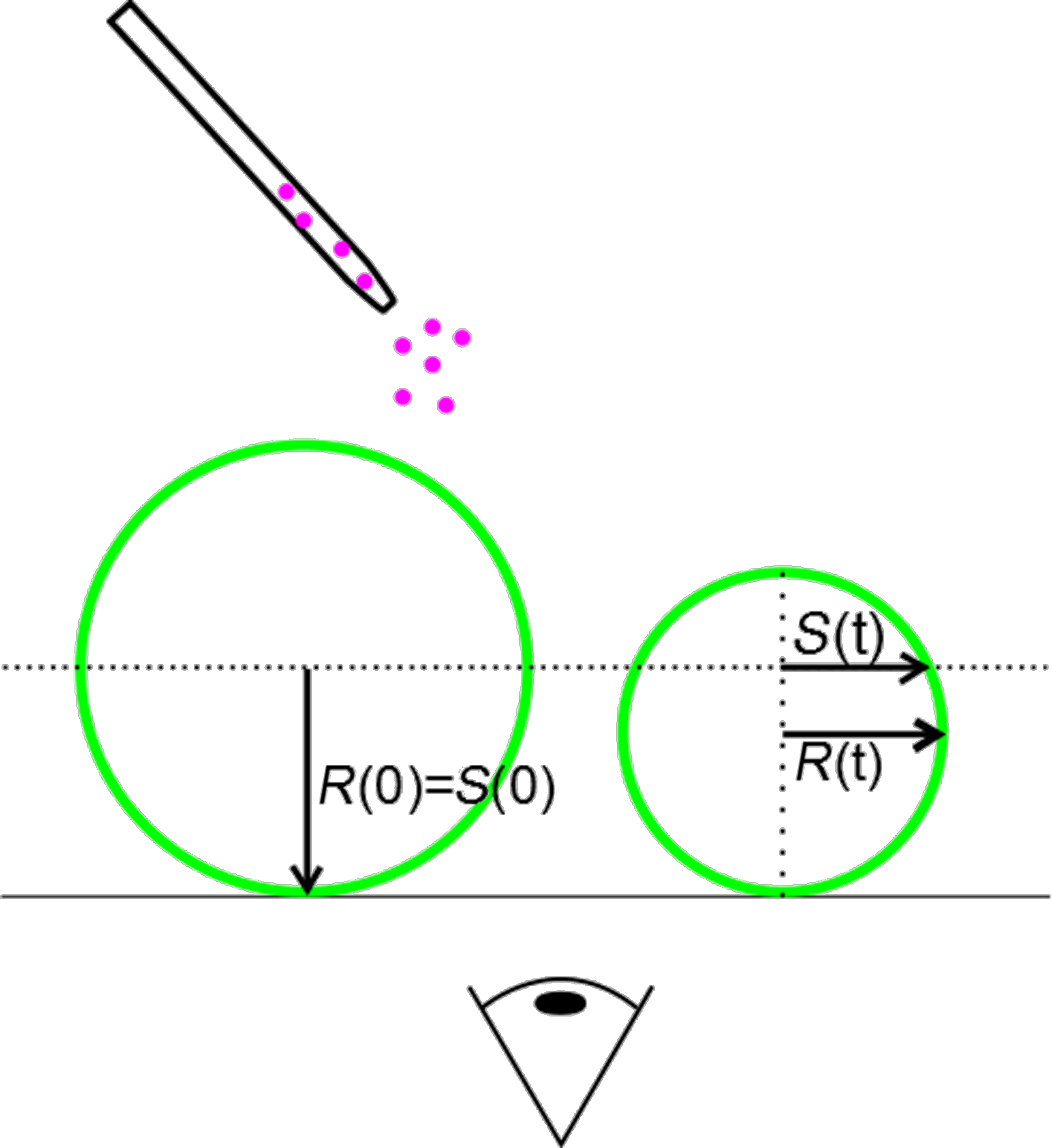
Nanoparticles are added to the medium. After equilibration a vesicle is observed at constant focal height. The evolution of the vesicle size is monitored by means of the observed cross section area *S*(*t*).

Figure 9 describes the easy experimental procedure. Due to the slight density difference between inner and outer medium the GUVs sink down to the chamber bottom. Vesicles in the fluid state will show strong adhesion to the cover glass, rupture and form a solid supported bilayer (SLB) eventually. However, vesicles settling down on top of such a SLB show no significant adhesion and are used for the experiment. This procedure is not necessary in the case of gel-phase vesicles. A suitable GUV, i.e., an isolated unilamellar vesicle without membrane inclusions, was chosen for further examination by fluorescence microscopy (Zeiss Axiovert 200M). The nanoparticles dispersed in glucose medium were applied to the chamber from above. The particle surface concentration was *C*_s_ ≈ 10 m^2^/L in all experiments. The surface area concentration denotes the integrated particle surface per volume as follows:

[8]
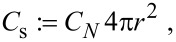


where *C_N_* is the number of particles per volume. The particles reach the vesicles by diffusive transport and after short equilibration the measurement is started.

The area of the vesicle cross section is analyzed at constant focal height and different time points. The analysis is performed with ImageJ. The actual surface area *A* of the vesicle can be easily calculated from the observed cross section area *A*_c_ (see Figure 9):

[9]
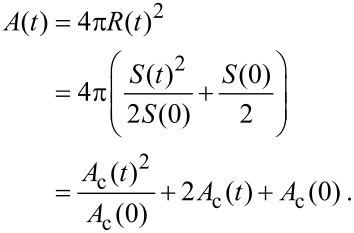


Assuming the particle concentration around the vesicle high enough to remain constant over time and assuming that particles cannot escape from the vesicle once they are internalized, the rate of particle uptake will be proportional to the surface area of the vesicle and the surface area concentration *C*_s_ of particles:

[10]
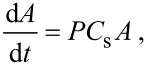


where *P* denotes an effective membrane “permeability”, i.e., its affinity to particle uptake. Hence, one would expect an exponential decay of the vesicle surface area with a decay constant *τ*^−1^ = *PC*_s_:

[11]
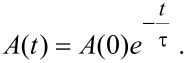


Fitting this equation to the experimental *A*(*t*)-curves yields the decay constants in Table 1.

## References

[1] Lu X, Liu Y, Kong X, Lobie P E, Chen C, Zhu T (2013). Small.

[2] Casals E, Gonzalez E, Puntes V F (2012). J Phys D: Appl Phys.

[3] Fadeel B, Garcia-Bennett A E (2010). Adv Drug Delivery Rev.

[4] Boisselier E, Astruc D (2009). Chem Soc Rev.

[5] Murthy S K (2007). Int J Nanomed.

[6] Petros R A, DeSimone J M (2010). Nat Rev Drug Discovery.

[7] Wang T, Bai J, Jiang X, Nienhaus G U (2012). ACS Nano.

[8] Verma A, Uzun O, Hu Y, Hu Y, Han H-S, Watson N, Chen S, Irvine D J, Stellacci F (2008). Nat Mater.

[9] Iversen T-G, Skotland T, Sandvig K (2011). Nano Today.

[10] Jiang X, Dausend J, Hafner M, Musyanovych A, Röcker C, Landfester K, Mailänder V, Nienhaus G U (2010). Biomacromolecules.

[11] Kulkarni S A, Feng S-S (2013). Pharm Res.

[12] Chithrani B D, Ghazani A A, Chan W C W (2006). Nano Lett.

[13] Zhao Y, Sun X, Zhang G, Trewyn B G, Slowing I I, Lin V S-Y (2011). ACS Nano.

[14] Farsad K, De Camilli P (2003). Curr Opin Cell Biol.

[15] Zimmerberg J, Kozlov M M (2006). Nat Rev Mol Cell Biol.

[16] Helfrich W (1973). Z Naturforsch, C.

[17] Jülicher F (1996). J Phys II.

[18] Lipowsky R, Döbereiner H-G (1998). Europhys Lett.

[19] Marsh D (1990). Handbook of Lipid Bilayers.

[20] Dietrich C, Angelova M, Pouligny B (1997). J Phys II.

[21] Deserno M, Gelbart W M (2002). J Phys Chem B.

[22] Reynwar B J, Illya G, Harmandaris V A, Müller M M, Kremer K, Deserno M (2007). Nature.

[23] Šarić A, Cacciuto A (2012). Phys Rev Lett.

[24] Bahrami A H, Lipowsky R, Weikl T R (2012). Phys Rev Lett.

[25] Koltover I, Rädler J O, Safinya C R (1999). Phys Rev Lett.

[26] Roiter Y, Ornatska M, Rammohan A R, Balakrishnan J, Heine D R, Minko S (2008). Nano Lett.

[27] Jaskiewicz K, Larsen A, Schaeffel D, Koynov K, Lieberwirth I, Fytas G, Landfester K, Kroeger A (2012). ACS Nano.

[28] Le Bihan O, Bonnafous P, Marak L, Bickel T, Trépout S, Mornet S, De Haas F, Talbot H, Taveau J-C, Lambert O (2009). J Struct Biol.

[29] Ahmed S, Savarala S, Chen Y, Bothun G, Wunder S L (2012). Small.

[30] Westerhausen C, Strobl F G, Herrmann R, Bauer A T, Schneider S W, Reller A, Wixforth A, Schneider M F (2012). Biophys J.

[31] Simons K, Gerl M J (2010). Nat Rev Mol Cell Biol.

[32] El-Sayed A, Harashima H (2013). Mol Ther.

[33] Anderson T H, Min Y, Weirich K L, Zeng H, Fygenson D, Israelachvili J N (2009). Langmuir.

[34] Helfrich W, Lipowsky R, Sackmann E (1995). Tension-Induced Mutual Adhesion and a Conjectured Superstructure of Lipid Membranes. Handbook of Biological Physics.

[35] Leirer C, Wunderlich B, Myles V M, Schneider M F (2009). Biophys Chem.

[36] Leirer C T, Wunderlich B, Wixforth A, Schneider M F (2009). Phys Biol.

[37] Seto H, Yamada N L, Nagao M, Hishida M, Takeda T (2008). Eur Phys J E.

[38] Dimova R, Pouligny B, Dietrich C (2000). Biophys J.

[39] Hamada T, Morita M, Miyakawa M, Sugimoto R, Hatanaka A, Vestergaard M C, Takagi M (2012). J Am Chem Soc.

[40] Israelachvili J N (1992). Intermolecular and Surface Forces.

[41] Helfrich W (1994). Prog Colloid Polym Sci.

[42] Barneveld P A, Scheutjens J M H M, Lyklema J (1992). Langmuir.

[43] Franke T, Leirer C T, Wixforth A, Dan N, Schneider M F (2009). ChemPhysChem.

[44] Döbereiner H G, Käs J, Noppl D, Sprenger I, Sackmann E (1993). Biophys J.

[45] Reynwar B J, Deserno M (2011). Soft Matter.

[46] Chernomordik L V, Zimmerberg J, Kozlov M M (2006). J Cell Biol.

[47] Shang L, Nienhaus K, Nienhaus G U (2014). J Nanobiotechnol.

[48] Cho E C, Xie J, Wurm P A, Xia Y (2009). Nano Lett.

[49] Bondar O V, Saifullina D V, Shakhmaeva I I, Mavlyutova I I, Abdullin T I (2012). Acta Naturae.

[50] Lunov O, Zablotskii V, Syrovets T, Röcker C, Tron K, Nienhaus G U, Simmet T (2011). Biomaterials.

[51] Gao H, Shi W, Freund L B (2005). Proc Natl Acad Sci U S A.

[52] Maniti O, Blanchard E, Trugnan G, Lamazière A, Ayala-Sanmartin J (2012). Int J Biochem Cell Biol.

[53] Römer W, Berland L, Chambon V, Gaus K, Windschiegl B, Tenza D, Aly M R E, Fraisier V, Florent J-C, Perrais D (2007). Nature.

[54] Heimburg T, Jackson A D (2007). Biophys J.

[55] Angelova M I, Soléau S, Méléard P, Faucon F, Bothorel P, Helm C, Lösche M, Möhwald H (1992). Preparation of giant vesicles by external AC electric fields. Kinetics and applications. Trends in Colloid and Interface Science VI.

[56] Blechinger J, Herrmann R, Kiener D, García-García F J, Scheu C, Reller A, Bräuchle C (2010). Small.

